# A Comparative Full-Length Transcriptome Analysis Using Oxford Nanopore Technologies (ONT) in Four Tissues of Bovine Origin

**DOI:** 10.3390/ani14111646

**Published:** 2024-05-31

**Authors:** Xinyue Liu, Jiaxin Wu, Meichen Li, Fuyuan Zuo, Gongwei Zhang

**Affiliations:** 1 College of Animal Science and Technology, Southwest University, Rongchang, Chongqing 402460, China; luckyliuxy@163.com (X.L.); wjiaxin0225@163.com (J.W.); limeichen1998@163.com (M.L.); zfuyuan@163.com (F.Z.); 2 Beef Cattle Engineering and Technology Research Center of Chongqing, Southwest University, Rongchang, Chongqing 402460, China

**Keywords:** alternative splicing, APA, cattle, ONT, TEST-specific genes, transcription factors

## Abstract

**Simple Summary:**

A comparative transcriptomic analysis using Oxford Nanopore Technologies (ONT) was conducted in bovine testes (TESTs), ovaries (OVAs), muscles (MUSCs), and livers (LIVs). Once the samples (*n* = 18) were analyzed, TESTs exhibited the most alternative polyadenylation (APA) events related to male reproductive processes.

**Abstract:**

The transcriptome complexity and splicing patterns in male and female cattle are ambiguous, presenting a substantial obstacle to genomic selection programs that seek to improve productivity, disease resistance, and reproduction in cattle. A comparative transcriptomic analysis using Oxford Nanopore Technologies (ONT) was conducted in bovine testes (TESTs), ovaries (OVAs), muscles (MUSCs), and livers (LIVs). An average of 5,144,769 full-length reads were obtained from each sample. The TESTs were found to have the greatest number of alternative polyadenylation (APA) events involved in processes such as sperm flagellum development and fertilization in male reproduction. In total, 438 differentially expressed transcripts (DETs) were identified in the LIVs in a comparison of females vs. males, and 214 DETs were identified in the MUSCs between females and males. Additionally, 14,735, 36,347, and 33,885 DETs were detected in MUSC vs. LIV, MUSC vs. TEST, and OVA vs. TEST comparisons, respectively, revealing the complexity of the TEST. Gene Set Enrichment Analysis (GSEA) showed that these DETs were mainly involved in the “spermatogenesis”, “flagellated sperm motility”, “spermatid development”, “reproduction”, “reproductive process”, and “microtubule-based movement” KEGG pathways. Additional studies are necessary to further characterize the transcriptome in different cell types, developmental stages, and physiological conditions in bovines and ascertain the functions of the novel transcripts.

## 1. Introduction

Cattle (*Bos taurus*) hold significant economic importance worldwide, and many studies have been conducted to improve or enhance productivity and reduce disease susceptibility [1]. The testis (TEST) is an essential and complex reproductive organ composed of various somatic and germ cells, which interact to facilitate the development of the TEST and functional spermatogenesis. Compared with somatic organs, such as the liver (LIV) and muscles (MUSCs), besides basic cellular life processes, the TEST has highly specific and complex physiological processes closely related to male fertility, including spermatogenesis. The complexity of cellular composition and function in the TEST is reflected in the diversity of the transcripts expressed in this tissue. These transcripts encode a wide range of proteins and non-coding RNAs, including those involved in spermatogenesis, steroidogenesis, and cell signaling. The complexity of the TEST transcriptome is further reflected in the fact that many transcripts are unique to the TEST [2,3]. These TEST-expressed genes (*TEX*) are likely to play essential roles in the specialized functions of the TEST [4,5], such as cell differentiation, germ cell development, and hormone-based regulation. The exploration of new transcripts and variable splicing of genes with high expression and important physiological functions in the TEST [6,7,8,9,10] is of great significance for further understanding the complex regulation of male reproductive function.

Some of the most studied TEST-specific genes (TSGs) are the Y-linked gene family, which includes sex-determining region Y (SRY) and several other Y chromosome-specific genes. SRY plays a vital role in determining male sex by initiating the differentiation of the gonadal primordium into a TEST [6]. Other Y-linked genes, such as *DAZ* (deleted in azoospermia), are implicated in spermatogenesis [7]. Another significant family of TSGs includes those encoding protamines. Protamines are small, Arg-rich proteins that replace histones during the later stages of spermatogenesis, allowing for the extreme condensation of the DNA in sperm [8]. Protamine levels are associated with male infertility. The TEST-specific protein Y-encoded (TSPY) is another TSG implicated in the manifestation of testicular germ cell tumors. TSPY is thought to promote cell proliferation and inhibit apoptosis, contributing to tumorigenesis [9]. TEST-specific serine kinases represent another group of TSGs. The proteins encoded by the mutants of these TSGs are involved in spermatid differentiation and associated with male infertility [10].

Oxford Nanopore Technologies (ONT) sequencing has revolutionized transcriptomic studies by offering longer read lengths, real-time data generation, and direct RNA sequencing capabilities [11]. The technology of ONT sequencing works by detecting the changes in electrical conductivity generated as DNA or RNA molecules pass through a protein nanopore. The resulting signal is decoded into a sequence of nucleotides [12]. ONT sequencing has been used for transcriptomics-based investigation of various aspects of domesticated animals. For example, it has been used to study the transcriptomic complexity in pigs (*Sus scrofa*) [13] and identify the genes involved in follicle selection in chickens [14]. Until recently, transcriptome annotations, including the bovine genome, were primarily based on short-read RNA-seq data obtained using next-generation sequencing platforms. Only a few studies have focused on the ONT-based sequencing of bovine transcriptomes, such as a recent study that used ONT to characterize the poll allele in Brahman cattle [15]. Another study demonstrated the power of long-read sequencing for transcriptome annotation by coupling ONT with large-scale multiplexing of 93 samples comprising 32 tissues collected from adult male and female Hereford cattle [14]. Of these, >7000 transcript isoforms were extremely tissue specific, and 61% of these were attributed to the TEST, which exhibited the most complex transcriptome of all the analyzed tissues [16].

However, a comprehensive annotation of transcript isoforms in Chinese cattle is lacking. The potential of ONT sequencing in cattle transcriptomics is vast. Studying the TEST-related transcriptome is a vital area of research that can provide a better understanding of the biology of this organ in cattle. According to data from the United States Department of Agriculture (USDA), in 2020, there were a total of eight countries and regions worldwide with beef production exceeding one million tons. The United States produced 12.4 million tons of beef, Brazil produced 10.1 million tons, the European Union produced 7.8 million tons, and China produced 6.8 million tons, ranking fourth. China is also the only Asian country among the top eight producers. Therefore, beef production is crucial for China. Bashan cattle are mainly distributed in the mountainous area of China’s Daba Mountains. Bashan cattle are highly suitable for breeding, with a tolerance to rough feeding and strong disease resistance, which traits were strictly selected and bred locally [17]. Bashan cattle originally served as draft animals for plowing but are gradually transitioning to meat production, playing a significant role in increasing income for mountainous farmers. Due to their relatively small size and lower meat yield, the population of Bashan cattle has been declining. They exhibit non-seasonal reproduction. Through the analysis of multi-tissue full-length transcriptomes, we aim to gain a better understanding of the genetic potential of this breed. Through our research, we aim to obtain comprehensive transcriptome sequencing data from the LIV, MUSC, TEST, and ovary (OVA) tissues of Bashan cattle. We hypothesize that transcriptome complexity varies across different tissues of Bashan cattle. Compared to the LIV (primarily involved in metabolism) and MUSCs (primarily used for meat production), the TEST (which contains both somatic and germ cells) is likely to exhibit numerous specifically expressed genes and transcripts. These differentially expressed genes and transcripts may be closely related to male reproductive processes, such as androgen metabolism and spermatogenesis. Prediction of testicular highly expressed transcripts and alternative splicing in this study may facilitate selection programs seeking to improve productivity traits, fertility, and environmental adaptation factors of considerable scientific and economic interest for cattle. The findings of this study regarding TEXs could also help the development of new methods for treating male infertility and other testicular disorders.

## 2. Materials and Methods

### 2.1. Animals and Sample Collection

LIV, MUSC, OVA, and TEST tissue samples were collected from three male and three female Bashan cattle, all aged 20–30 months old. For males, LIV, MUSC, and TEST tissue samples were obtained from each of the cattle (*n* = 3, 9 samples in total). For females, LIV, MUSC, and OVA tissue samples were obtained from each of the cattle (*n* = 3, 9 samples in total). The sampling time was January, 2023. The females were heifers, and the complete ovary was sampled for RNA extraction. The MUSC was taken from the longissimus dorsi MUSC, while the TEST was cut from the vertical axis and a small piece of tissue in the middle was taken. All samples were collected from a local slaughterhouse. All experimental procedures were approved by the guidelines established by the Institutional Animal Care and Use Ethics Committee of Southwest University (IACUC-20240506-01). Samples were collected within 30 min post-euthanasia, flash-frozen in liquid nitrogen, and stored at −80 °C until processing.

### 2.2. RNA Extraction and cDNA Library Construction

RNA extraction and cDNA library construction adhered to the standard protocol provided by ONT and were supported by Biomarker Technologies Co. Ltd. (Beijing, China). Total RNA extraction was conducted utilizing TRIzol kits from Solarbio LIFE SCIENCE (Beijing, China). Subsequently, the Nanodrop2000 (Waltham, MA, USA) was employed to determine nucleic acid concentrations and purity, while integrity was confirmed using the Agilent 2100 Bioanalyzer (Palo Alto, CA, USA) and the LabChip GX (Waltham, MA, USA). For library construction, Poly (A) mRNA was initially purified from total RNA using mRNA capture beads from Vazyme (Nanjing, China). Reverse transcription and double-stranded DNA amplification were conducted sequentially with reverse transcription and amplification primers. NEBNext FFPE DNA Repair Mix and the NEBNext Ultra II End Repair/dA Tailing Module were then utilized to perform damage repair and end repair with the addition of nucleic acid fragments. Finally, sequencing adapter ligation was carried out using the Ligation Sequencing Kit 1D (PM) (SQK-LSK109) from Nanopore (Oxford, UK).

Following a quality inspection of the library (concentration X > 2 ng/μL), Flow Cell Priming mix was prepared using a sequencing chip preparation kit (EXP-FLP001 PRO.6) (Oxford, UK). Subsequently, the library was prepared using the Ligation Sequencing Kit 1D (PM) (SQK-LSK109) (Oxford, UK). Finally, the MinKnow software (Version 2.2) was operated on the PromethION48 sequencer (Oxford, UK) to initiate sequencing with the PromethION Flow Cells (FLO-PRO002) (Oxford, UK) chip, running with a default time of 72 h.

### 2.3. ONT-Based Long-Read Processing

Raw reads underwent initial filtering, retaining reads with a minimum average quality score of six and a minimum length of 350 bp. Ribosomal RNA sequences were discarded after mapping to the rRNA database. Full-length non-chimeric (FLNC) transcripts were identified by searching for primers at both ends of the reads. Clusters of FLNC transcripts were obtained after mapping to the reference genome, ARS-UCD1.2, using Minimap2 (Version 2.16) [18]. Consensus isoforms were derived by filtering within each cluster using Pinfish (Version 0.1.0). Mapped reads were further condensed using the cDNA_Cupcake package (Version 5.80), considering a minimum coverage of 85% and a minimum identity of 90%. Redundant transcripts were condensed without considering a 5′ difference.

### 2.4. Identification of Fusion Transcripts

Firstly, full-length transcriptomes were obtained through sequencing and analyses. The analytic process for obtaining a full-length transcriptome mainly includes three stages: full-length sequence recognition, full-length sequence polishing to obtain consistent sequences, and redundancy removal for consistent sequences. The detailed steps were as follows: (1) Filter low-quality (length less than 200 bp, Q score less than 6) sequences and ribosomal RNA sequences from the original downstream sequence and obtain the full-length sequence based on the presence of primers at both ends of the sequence. (2) Polish the full-length sequence obtained from the previous step to obtain a consistent sequence. (3) Perform fusion transcript screening on each sample using the consistent sequence before redundancy removal under the following conditions. Candidate fusion transcripts were identified based on the following criteria: (1) mapping to ≤2 loci, (2) minimum coverage of 5% and ≥1 bp for each locus, (3) total coverage ≥ 95%, and (4) loci distance ≥ 10 kb.

### 2.5. Structural Analyses

Transcript annotations were validated against known reference transcripts using GffCompare (Version, 0.12.6, “https://ccb.jhu.edu/software/stringtie/gffcompare.shtml, (accessed on 5 July 2023)”. Alternative splicing (AS) events, including retained introns (RIs), exon skips (ESs), alternate donor sites (ADs), alternate acceptor sites (AAs), and mutually exclusive exons (MEEs), were identified using the AStalavista tool (Version 1.0). Simple sequence repeats (SSRs) in the transcriptome were identified using MISA (Version 1.0). APA analysis was conducted using TAPIS (Version 1.2.1) [19], and CDSs were predicted using TransDecoder (Version, 3.0.0, “https://github.com/TransDecoder/TransDecoder (accessed on 10 July 2023)”.

### 2.6. Prediction of Transcription Factors (TFs)

Animal TFs were identified using AnimalTFDB (Version 1.6). Putative protein-coding RNAs were filtered based on minimum length and exon number thresholds. 

### 2.7. Gene Functional Annotation

Firstly, sequence alignment was performed for the reference genome to obtain known transcripts/genes. The remaining sequences were subjected to variable splicing analyses to obtain new transcripts/genes. The obtained new transcript/gene sequences were then compared with the NR [20], Swissprot [21], GO [22], COG [23], KOG [24], Pfam [25], and KEGG [26] databases to obtain annotation information.

### 2.8. Quantification of Gene/Transcript Expression Levels and Differential Expression Analyses

In the current study, we utilized full-length sequencing transcriptomes aligned against known transcriptomes from the genome as a reference for sequence alignment and subsequent analyses. We employed minimap2 to align full-length sequences with known transcripts of the reference genome, obtaining correspondence information of the transcripts. The reference genome used was Bos_taurus, and the version ARS_UCD1.3 “https://www.ncbi.nlm.nih.gov/datasets/genome/GCF_002263795.2/ (accessed on 10 July 2023)”, was employed. Statistical results of the comparison of full-length reads and the known transcriptome are presented in Table S6. Full-length reads were mapped to the known transcriptome sequences, with reads having a match quality of >5 being used for quantification. Expression levels were estimated as reads per gene/transcript per 10,000 mapped reads.

To ensure that the number of fragments accurately reflected transcript expression levels, it was necessary to normalize the number of mapped reads in the samples. The counts per million (CPM) method was used as an indicator of transcript or gene expression levels. The CPM was calculated using the following formula (“reads mapped to transcript” represents the number of reads aligned to a specific transcript; “total reads aligned in sample” represents the total number of fragments aligned to the known transcriptome from the genome): CPM = (reads mapped to transcript/total reads aligned in sample) × 1,000,000.

Differential expression analyses of the two groups were performed using the DESeq2 R package (Version 1.6.3). DESeq2 [27] employs statistical routines based on the negative binomial distribution to determine differential expression in digital gene expression data. The resulting *p* values were adjusted using Benjamini and Hochberg’s approach to control the false discovery rate. During the detection of differentially expressed transcripts, fold changes ≥ 1.5 and *p* values < 0.01 were used as screening criteria. The fold change represented the ratio of expression levels between two sample groups. The *p* value served as the significance indicator for screening differentially expressed genes. Genes that show significant differences in expression levels under different conditions are called differentially expressed genes (DEGs). Different transcripts refer to different mRNA variants transcribed from the same gene, and transcripts with significant differences in expression levels are called differentially expressed transcripts (DETs).

### 2.9. Functional Enrichment Analyses

GO enrichment analyses of DEGs were performed using the GOseq R package (version 1.24.0), which adjusts for gene length bias in DEGs using the Wallenius non-central hypergeometric distribution [28]. KEGG pathway enrichment analyses were conducted using KOBAS (Version 3.0) [29] software, testing the statistical enrichment of DEGs in the KEGG pathways.

### 2.10. Protein–Protein Interaction (PPI) Analyses

DEG sequences from different tissues were aligned with the genome using BLASTx. Predicted PPIs of proteins encoded by these DEGs were obtained from the STRING database “http://string-db.org/ (accessed on 12 July 2023)”. These PPIs were visualized using Cytoscape (Version 3.10.1).

### 2.11. TF–Gene Interaction Network Analyses Were Performed Using the Network Analyst Tool and the JASPAR Database

Briefly, we first uploaded the gene list to the Network Analyst website “https://www.networkanalyst.ca/ (accessed on 15 July 2023)”, then selected the corresponding species and assigned the analytic category as TF–gene interactions, and finally selected the JASPAR database “https://jaspar.elixir.no/ (accessed on 15 July 2023)”for transcription factor prediction.

## 3. Results

### 3.1. Alternative Splicing Structural Analyses of Bovine Tissues

**Figure 1:**
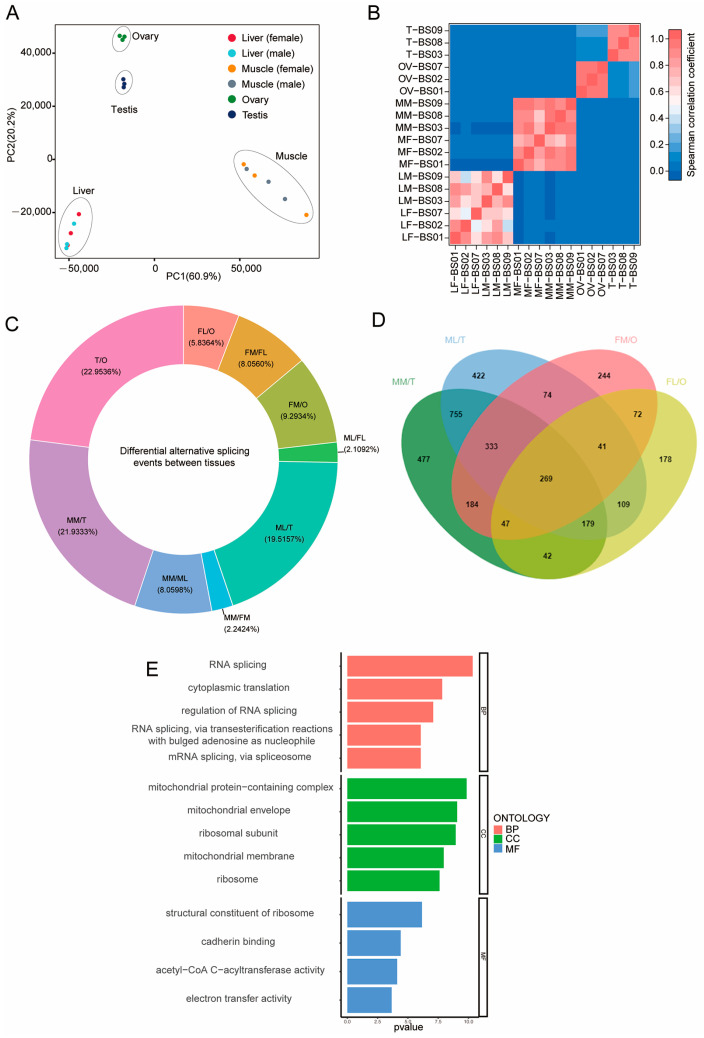
Alternative splicing events identified by ONT. (**A**) PCA analysis of 18 samples. (**B**) Spearman cluster analysis of transcript expression of 18 samples. (**C**) The differential alternative splicing events for each tissue type in the cattle. (**D**) A Wayne diagram showing genes with alternative splicing events for each tissue type in the cattle. (**E**) The differential alternative splicing genes in TEST and OVA samples were enriched in biological processes, including RNA splicing, cytoplasmic translation, and molecular functions of structural constituents of ribosomes, cadherin binding, and electron transfer activity, among others. MM: male MUSC; ML: male LIV; FM: female MUSC; FL: female LIV; O: OVA; T: TEST.

The transcriptomes of 18 samples collected from four tissue types were obtained by ONT sequencing (Table S1). Identification of 4,198,897–6,416,610 full-length sequences using valid sequencing data yielded 4,669,243–6,931,917 clean reads with an average read quality score > 7 and a length > 50 bp (Table S2). The full-length sequence was filtered to obtain a consensus isoform, and redundancy analysis was performed after all the consensus isoforms were compared to the reference genome, due to which 114,316 transcripts were finally obtained. The principal component analyses (Figure 1A) and correlation analyses of gene expression for the 18 samples (Figure 1B) indicated similar expression patterns in the same tissues. In addition, 43,325 novel transcripts were functionally annotated according to nine databases (Table S3).

Among the five alternative splicing types prevalent in the tissues, alternative exon skipping accounted for a maximum of 54.13–67.55% and mutually exclusive exons accounted for a minimum of 1.88–4.54% for the 18 samples (Table S4). 

Differential splicing events were present in various organs. Regardless of gender, the differential splicing between reproductive and non-reproductive organs was significantly higher than that observed among non-reproductive organs. And the largest quantities of differential alternative splicing events were observed between the TEST and OVA samples (Figure 1C). This suggested the complexity and diversity of ovarian and testicular functions, as they were responsible for crucial reproductive functions and gamete production, with gene splicing variations reflecting the intricacy and diversity of gene functions. Moreover, compared to TESTs, the number of differential splicing genes in male LIVs and MUSCs was 2182 and 2286, respectively, with a common set of 1536 differential splicing genes. These variations in splicing may be associated with specific functions in the TESTs (Figure 1D). In contrast, compared to OVAs, female LIVs and MUSCs had 937 and 1264 differential splicing genes, respectively, with a common set of 429 differential splicing genes likely related to specific functions in the OVAs (Figure 1D). There were 269 common differential splicing genes between TESTs and MUSCs/LIVs, as well as OVAs and MUSCs/LIVs (Figure 1D). The splicing variations in these genes between TESTs and OVAs suggested their potential key roles in the unique reproductive functions of the TEST and the OVA, possibly involving meiosis, a shared physiological process in the generation of sperm and oocytes. The biological processes implicated include RNA splicing, cytoplasmic translation, and molecular functions of structural constituents of ribosomes, cadherin binding, and electron transfer activity, among others (Figure 1E). Additionally, fusion transcripts were predicted for the consistent transcripts obtained, and 18 samples obtained fusion transcripts ranging from 21 to 144. SSR prediction was performed on the transcripts of all samples without redundancy. Six types of nucleotides—mono-, di-, tri-, tetra-, penta-, and hexa-nucleotides—and compound SSRs were identified, and 65,661 SSRs were obtained (Table S5). The Animal TFDB 3.0 [30] database was used to identify the animal-specific transcription factors (TFs). A total of 6044 TFs were predicted from the new transcripts identified in this study. 

### 3.2. APA Analyses of Bovine Tissues

**Figure 2:**
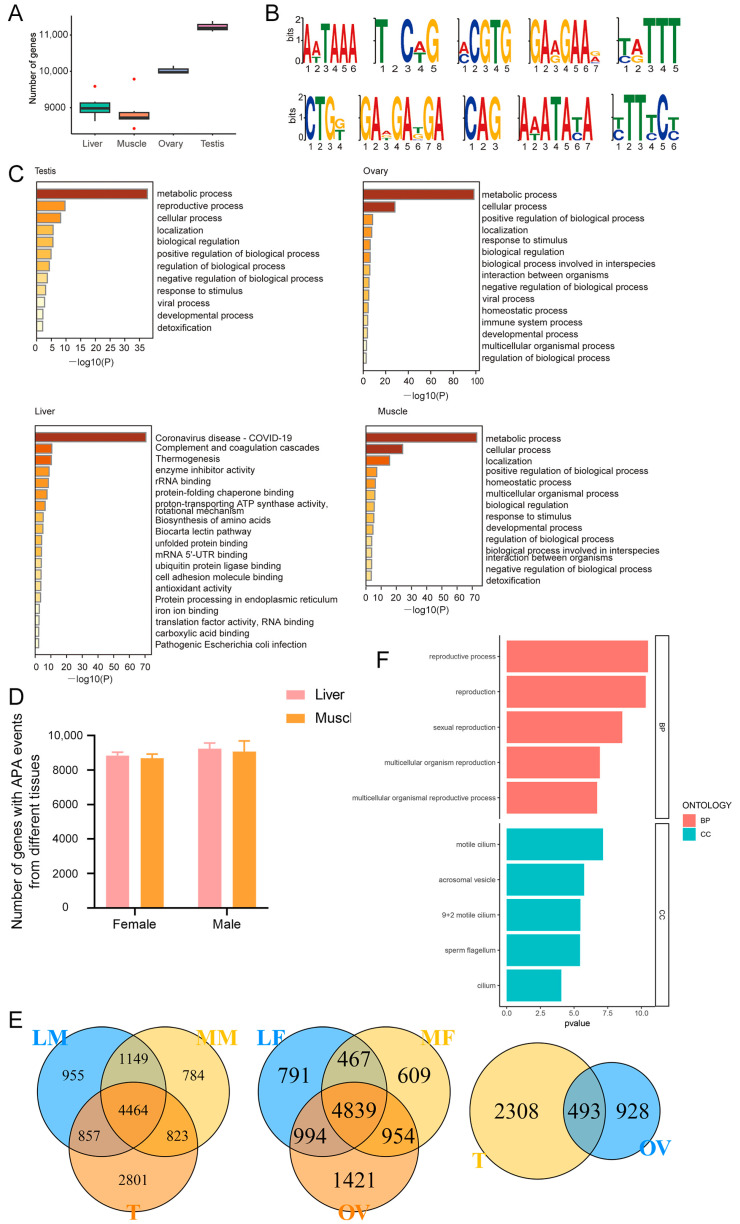
Schematic features of APA. (**A**) Number of genes with APA. (**B**) Top ten motifs of APA. (**C**) Functional analyses of top 200 highest-number-of-APA-event genes. (**D**) The numbers of APA genes in the LIVs and MUSCs between the females and males were similar. (**E**) Differential APA gene analyses for each tissue in cattle. LM, male liver; MM, male muscle; LF, female liver; MF, female muscle; OV, ovary; T, testis. (**F**) Pathway analyses revealed that the 2308 TEST-specific genes were significantly enriched in male reproductive processes, with associated cellular components including motile cilium, acrosome vesicle, “9 + 2” motile cilium, sperm flagellum, and cilium, which are related to sperm flagellum assembly and fertilization function.

Polyadenylation refers to the covalent linking of polyadenylate with messenger RNA (mRNA) molecules. In the process of protein biosynthesis, this is part of the way in which mature mRNA is produced ready for translation. In eukaryotes, polyadenylation is a mechanism by which mRNA molecules are interrupted at their 3′ end. The polyadenylate tail (or polyA tail) protects mRNA from exonuclease attacks and is important for transcription termination, mRNA export from the nucleus, and translation. APA (APA) of precursor mRNAs may contribute to transcriptomic diversity, genomic coding capacity, and gene regulatory mechanisms. We used the TAPIS pipeline to identify APA [19]. The results showed that the TESTs had the greatest number of genes with APA. The numbers of APA genes in the OVAs and TESTs were higher than those in the LIVs and MUSCs, while the numbers of APA genes in the LIVs and MUSCs of the females and males were similar (Figure 2A,D). The top ten motifs of APA are shown in Figure 2B. Signals in the pre-mRNA were recognized by core polyadenylation proteins. Several signals in the pre-mRNA direct the cleavage and polyadenylation (hereinafter, “polyadenylation”) machinery to a site. The sequence AATAAA (or something similar) is called the polyadenylation signal and is generally found 15–30 bases upstream of the site of cleavage [31,32]. To explore the function of genes with APA in the tissues, we focused on the top 200 genes with the highest APA aligned reads in each tissue. The functional annotation of the genes in TESTs showed that these genes were mainly involved in “reproductive process”, “metabolic process”, and “cellular process” processes, and so on (Figure 2C). Genes with high APA in OVAs were mainly related to “response to stimulus”, “metabolic process”, “positive regulation of biological process”, and “homeostatic process” processes.

Furthermore, we investigated the APA genes among different tissues. In males, there were 2801 TEST-specific APA genes compared to LIV and MUSC tissues (Figure 2E, left). In females, there were 1421 OVA-specific APA genes compared to LIV and MUSC tissues (Figure 2E, middle). When comparing TESTs and OVAs, there were 493 common APA genes, while TEST- and OVA-specific APA genes numbered 2308 and 928, respectively (Figure 2E, right). Pathway analyses revealed that the 2308 genes are significantly enriched in reproductive processes, with associated cellular components including the motile cilium, the acrosome vesicle, the “9 + 2” motile cilium, the sperm flagellum, and the cilium, which are related to sperm flagellum assembly and fertilization function (Figure 2F). In total, TESTs had the highest complexity in terms of APA.

### 3.3. DETs and DEGs among the Various Tissues Indicated the Complexity of Testicular Expression

**Figure 3:**
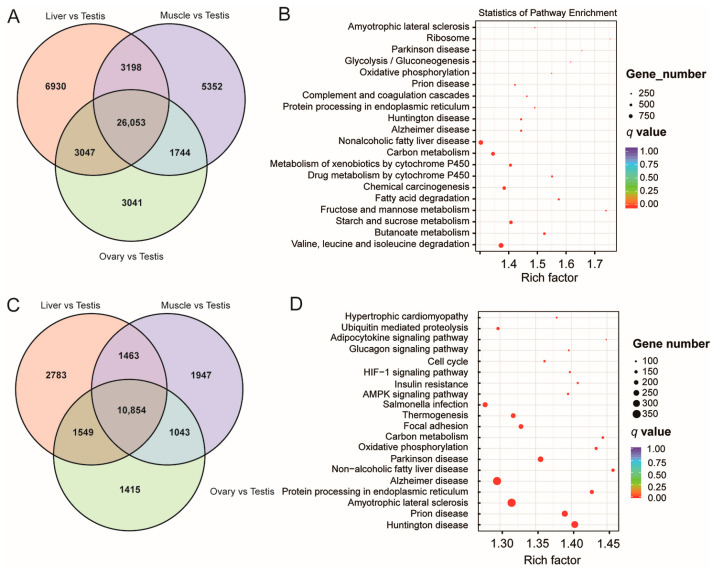
Analyses of differentially expressed transcripts (DETs) and genes (DEGs) for each tissue type vs. TESTs in cattle. (**A**) Venn diagram indicating the number of DETs identified through comparisons between LIVs and TESTs, MUSCs and TESTs, and OVAs and TESTs in cattle, respectively. (**B**) KEGG analysis diagram of the DETs that overlapped in the three comparisons. (**C**) Venn diagram indicating the number of DEGs identified by comparisons of LIVs and TESTs, MUSCs and TESTs, and OVAs and TESTs in cattle. (**D**) KEGG analysis diagram of the overlapping DEGs identified in the three comparisons.

The full-length sequencing transcriptome was mapped to the known transcriptome of the genome and used for subsequent analyses (Table S6). The full-length sequence was compared with the known transcriptome of the genome using Minimap2 to obtain the corresponding transcriptomic information. DESeq2 was then used to detect the DETs between the two groups [18]. The same tissues in the females and the males showed the fewest DETs (FDR ≤ 0.01; FC ≥ 2). However, the highest numbers of DETs were always identified in the comparisons between each tissue type and the TESTs (Table S7), indicating the complexity of the TESTs. Conversely, 26,053 overlapping DETs were identified among the following comparisons: LIVs vs. TESTs, OVAs vs. TESTs, and MUSCs vs. TESTs (Figure 3A).

Annotation based on the CGO, eggNOG, Gene Ontology (GO), KOG, and Kyoto Encyclopedia of Genes and Genomes (KEGG) databases was conducted. In the CGO database annotation, most genes were involved in “Posttranslational modification, protein turnover, chaperones” (1198 genes, ~12.47%) (Figure S1A); in the eggNOG database annotation, which indicated the complexity of the TESTs, most genes were enriched in “Function unknown” (5528 genes, ~33.29%) (Figure S1B); in the KOG database annotation, ~16.67% (5027) genes were annotated in “General function prediction” (Figure S1C).

The molecular functions of the GO class annotation indicated that these transcripts were involved in “binding”, “catalytic activity”, “molecular function regulator”, etc. (Figure S2A). The functional annotation of the KEGG class indicated that the DETs mainly participated in “Cellular Processes”, “Environmental Information Processing”, “Genetic Information Processing”, “Human Diseases”, “Metabolism”, and “Organismal Systems” (Figure S2B). These metabolic pathways especially included “Fatty acid degradation”, “Fructose and mannose metabolism”, “Starch and sucrose metabolism”, “Butanoate metabolism”, and “Valine, leucine, and isoleucine degradation” (Figure 3B). In addition, in terms of “Organismal Systems” pathways, 569 DETs (3.11%) were involved in “Thermogenesis”, which is related to a vital function of the TEST. The enriched GO terms of the DETs were mainly associated with “Structural molecule activity”, “Transporter activity”, and “Binding”, especially “Structural constituent of ribosome” (Figure S3).

Additionally, in total, 291 DEGs, 110 upregulated and 181 downregulated, were identified in the LIVs of the females and the males with respect to the significance criteria of |fold change| > 1.5 and *p* < 0.01 (Table S8), while 121 DEGs, 67 upregulated and 54 downregulated, were identified in the MUSCs of the females and the males, indicating a certain level of differential gene expression in the same tissues in the males and the females. Meanwhile, many DEGs (14,861) were detected in comparing the OVAs and TESTs. Additionally, 10,854 overlapping DEGs were identified in the comparisons of LIVs vs. TESTs, OVAs vs. TESTs, and MUSCs vs. TESTs (Figure 3C).

Annotation of these DEGs was also carried out based on the CGO, eggNOG, GO, KOG, and KEGG databases. Highly similar to the annotation results for DETs, the results for the annotation of DEGs indicated that most genes, 450 in number (11.95%), were involved in “General function prediction only” and that 422 genes (~11.21%) were involved in “Posttranslational modification, protein turnover, chaperones”, according to the CGO database (Figure S4A); a majority of the genes, numbering 721 (~38.97%), were enriched in “Function unknown” according to the eggNOG database, and a large number of genes had unexplored functions, which indicated the complexity of the TEST (Figure S4B); and 1978 genes (~17.68%) were enriched in “General function prediction” according to the KOG database (Figure S4C). The annotation for TFs revealed that most DEGs belonged to C2H2, HB-other, and bHLH TFs (Figure 3D).

Molecular functional annotation of the GO class indicated that these DEGs were also involved in “binding” terms, etc. (Figure S5A). Functional annotation of the KEGG class revealed that the DEGs were mainly involved in “Herpes simplex virus 1 infection” in “Human Diseases” pathways (Figure S5B). Meanwhile, the “PI3K-Akt signaling pathway” was the most enriched “Environmental Information Processing” pathway. Significantly, the DEGs were also enriched in the “HIF-1 signaling pathway”, revealing the high frequency of hypoxic responses in the TESTs (Figure 3D). The enriched GO terms of the DEGs were mainly related to “Structural molecule activity”, “Transporter activity”, and “Binding”, especially “Catalytic activity” (Figure S6).

**Figure 4:**
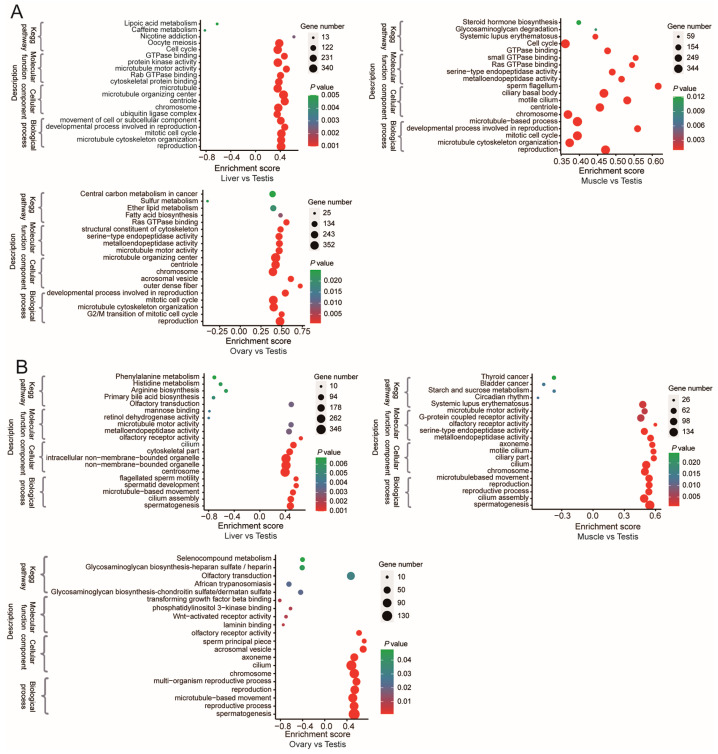
Gene Set Enrichment Analysis (GSEA) enrichment plots. (**A**) GSEA enrichment plots of GO terms and KEGG pathways comparing the DETs in the TEST vs. the other three tissues of the LIV, MUSC, and OVA. (**B**) GSEA enrichment plots of GO terms and KEGG pathways comparing the DEGs in the TEST vs. the other three tissues of the LIV, MUSC, and OVA.

Based on the assumption that the functions of genes were known, the pathway enrichment of the DETs and DEGs was conducted to predict their potential roles in the TEST using Gene Set Enrichment Analysis (GSEA). The DETs and DEGs were mainly involved in the “spermatogenesis”, “flagellated sperm motility”, “spermatid development”, “reproduction”, “reproductive process”, and “microtubule-based movement” KEGG pathways (Figure 4).

### 3.4. Testicular-Specific High-Expression Genes/Transcripts and Their Functional Annotation

**Figure 5:**
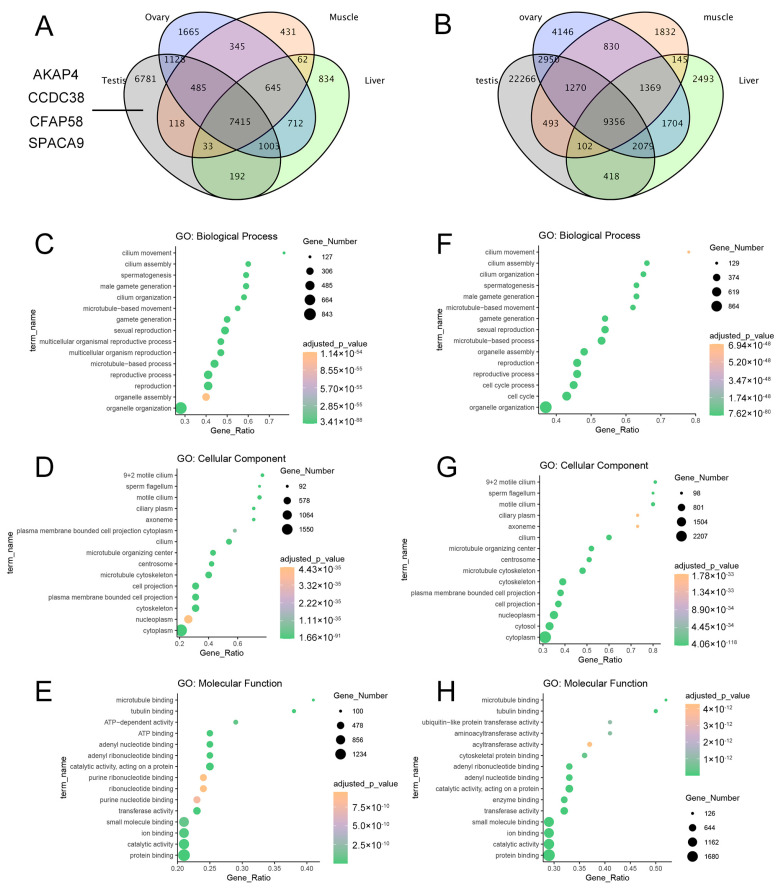
Pathway enrichment analyses of TEST specifically expressed genes/transcripts. (**A**) Venn diagram of genes expressed in different tissues. (**B**) Venn diagram of transcripts expressed in different tissues. (**C**–**E**) Biological process (**C**), cellular component (**D**), and molecular function (**E**) enrichment of TEST specifically expressed genes. (**F**–**H**) Biological process (**F**), cellular component, (**G**) and molecular function (**H**) enrichment of TEST specifically expressed transcripts.

The preceding data revealed the complexity of testicular gene expression and alternative splicing. To further elucidate the functional regulation and molecular pathways involved in TEST-specific gene expression and transcripts, we screened for highly expressed genes in TESTs and transcripts and conducted pathway enrichment analyses (fold change > 1.5, *p* < 0.01). Figure 5A depicts a Venn diagram of gene expression across the four tissues, with the numbers of genes specifically expressed in the LIVs, MUSCs, OVAs, and TESTs being 834, 431, 1665, and 6781, respectively. Meanwhile, in Figure 5B, the numbers of transcripts specifically expressed in the LIVs, MUSCs, OVAs, and TESTs are 2493, 1832, 4146, and 22,266, respectively. It was evident that the TESTs exhibited the highest numbers of genes and transcripts, further indicating the complexity of testicular function. GO pathway enrichment analyses revealed the 15 most significantly different pathways, with genes (Figure 5C–E) and transcripts (Figure 5F–H) exhibiting remarkably high similarity, being significantly enriched in biological processes closely related to spermatogenesis, such as reproduction, gamete generation, microtubule-based movement, cilium assembly, ciliary movement, and sperm development. Moreover, the cellular components were enriched in sperm flagella, “9 + 2” motile cilia, axonemes, microtubule organizing centers, centrioles, and microtubule cytoskeleton, indicating the importance of sperm development in testicular function. Molecular functions were involved in RNA binding, purine nucleotide binding, ATP binding, ATP-dependent activity, and microtubule binding, all of which were closely associated with sperm energy metabolism. Additionally, the results of the KEGG and Reactome pathway enrichment analyses are presented in Figure S7. These identified genes with testicular-specific expression included the CEP family, the *CFAP* family, the *CCDC* family, the *DNAH* family, the *IFT* family, the *TEKT* family, etc. Some of these genes have been proven to play key roles in mouse and human spermatogenesis through functional experiments (*AKAP4*, *CCDC38*, *CFAP58*, *SPACA9*, etc.), but there are still many genes whose functions have not been fully validated. Our study provides an expression profile of testicular-specific expression genes in cattle, and the study of these unknown genes is of great significance for deepening our understanding of male reproduction.

### 3.5. Expression of TEST-Specific Genes

To explore TEST-specific expressed genes, we next focused on the transcripts only expressed in TESTs (CPM > 0 in all 3 TEST samples, and CPM = 0 in the other 15 samples) compared with the other three tissues (LIV, MUSC, and OVA). As a result, 17,356 transcripts were detected (15,577 (89.75%) of them were novel transcripts), which corresponded to 8935 genes (5502 of them were novel genes).

**Figure 6:**
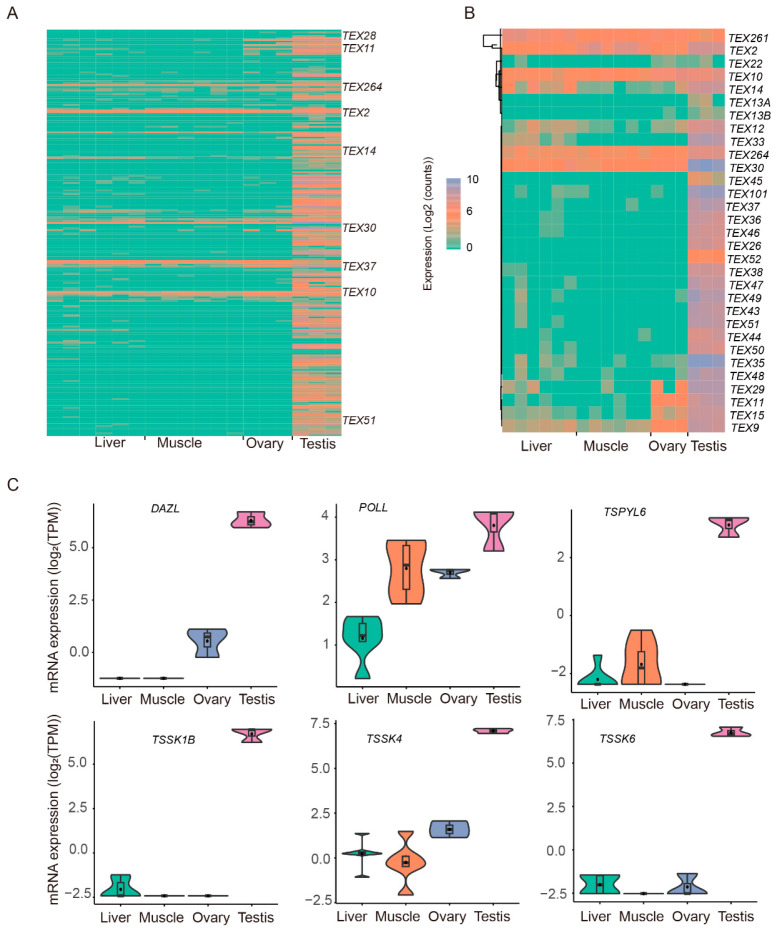
TEST-specific gene expression. Expression of all *TEX* transcripts (**A**) and *TEX* DEGs (**B**). Expression patterns of *TSSK1B*, *TSSK6*, *DAZL*, *TSPYL6*, *TSSK4*, and *POLL* (**C**).

In addition, among these genes, we focused on the *TEX* gene family. As a result, ONT sequencing detected the expression of 32 *TEXs* alongside 224 transcripts, 123 (54.91%) of which were novel. Of these genes, the most significant number of transcripts (33) were those of *TEX51*; the expression patterns of these transcripts (Figure 6A) and genes (Figure 6B) are shown. *TSSK1B*, *TSSK6*, *DAZL*, *TSPYL6*, *TSSK4*, and *POLL*, which were highly expressed in the TESTs, were differentially expressed in the TESTs and the three other tissues (Figure 6C). *TSSK1B* was significantly involved in the two GO terms “reproduction” and “reproductive process”, and *TSSK6* in seven GO terms, including “cell”, “cell part”, “reproduction”, “reproductive process”, “response to stimulus”, “multi-organism process”, and “biological regulation”. *DAZL* was involved in the terms “reproductive process”, “developmental process”, “growth”, and “multi-organism process”. *TSPYL6* was related to the terms “organelle” and “cell part”. *TSSK4* participated in the term “cellular process”. *POLL* was involved in the GO terms “organelle” and “cellular process”.

### 3.6. Identification of TEST Specifically Expressed Transcription Factors

**Figure 7:**
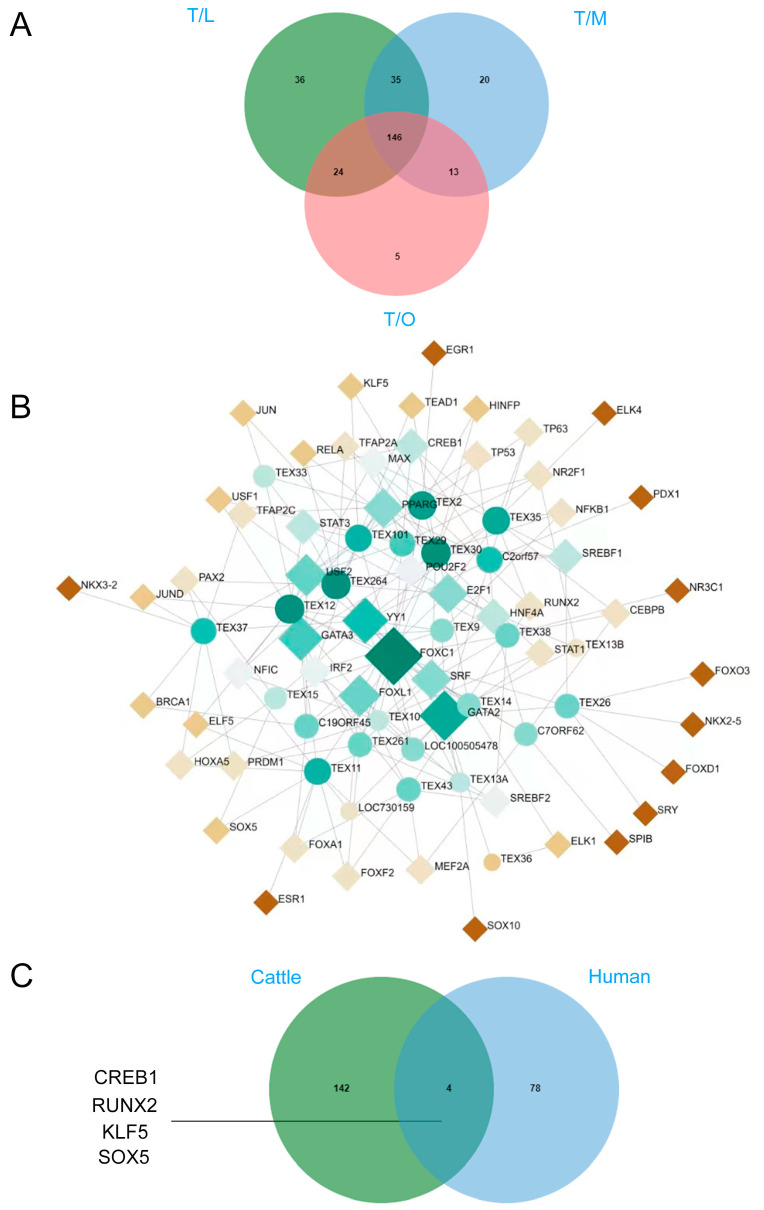
Identification of testicular-specific transcription factors. (**A**) Compared to the LIVs, MUSCs, and OVAs, 146 TEST specifically expressed transcription factors were predicted by ONT. (**B**) Predicted transcription factors of *TEXs* using the Network Analyst tool; 82 potential transcriptional regulatory factors were identified. (**C**) Transcription factors of *CREB1*, *RUNX2*, *KLF5*, and *SOX5* that might be involved in the regulation of *TEXs* were identified.

Additionally, according to previous studies which collectively identified 1253 hu-man transcription factors [33,34], we identified 1023 of them by ONT in our study. To further explore TEST-specific transcription factors, we intersected the transcription factors in the TESTs with those in the LIVs, MUSCs, and OVAs and obtained a total of 146 transcription factors that were considered highly reliable with respect to TEST-specific expression (Figure 7A). The pathway enrichment analyses for these transcription factors are shown in Figure S8. Additionally, we further illustrated the interactions among these transcription factors. As shown in Figure S9, these transcription factors also had complex interactions with each other. Considering the high expression of these proteins in the TEST, their mutual interactions are crucial for the regulation of their respective functions. Moreover, the proteins involved in these interactions might form protein complexes, playing pivotal roles in spermatogenesis and male reproductive regulation. This aspect is poised to become a key focus for future researchers studying such proteins. Furthermore, to delve deeper into the transcription factors involved in regulating *TEX* gene expression in the TEST, we employed the Network Analyst tool “https://www.networkanalyst.ca/ (accessed on 15 July 2023)” and the *JASPAR* database to predict the transcription factors (homo sapiens) for these 32 genes. Subsequently, we constructed a TF–gene interaction network, revealing that 82 transcription factors were implicated in the transcriptional regulation of *TEX* genes (Figure 7B). Following this, we then intersected these 146 transcription factors with the previously predicted 82, resulting in 4 transcription factors that were highly expressed in cattle TESTs, including *CREB1*, *RUNX2*, *KLF5*, and *SOX5* (Figure 7C). 

## 4. Discussion

### 4.1. TESTs Have the Greatest Number of Differential Alternative Splicing Events and Genes with APA Events

Before the commencement of this study, we hypothesized that, compared to the LIV and MUSC, the TEST would exhibit numerous specifically expressed genes and transcripts closely related to male reproductive processes, such as androgen metabolism and spermatogenesis. Furthermore, we speculated that these differentially expressed genes and transcripts might be closely associated with TEST-specific transcription factors and APA events. Excitingly, our findings have significantly supported this hypothesis. Over a billion cattle are raised for meat and dairy production worldwide. Although selection programs have significantly benefited from genomics tools in the past decade, a comprehensive characterization of the bovine transcriptome is essential to improve our understanding regarding the biological processes that underpin complex traits such as productivity, efficiency, and disease resistance, especially through analyzing the transcriptome using ONT sequencing [35,36,37,38,39]. TESTs are the male reproductive organs and play a critical role in sexual reproduction. Testicular growth and development are complex and strictly regulated processes [40]. Tissue-specific transcripts are fundamental for understanding the basis of the differences between tissues. They could serve as valuable biomarkers to explore economically important traits, as they are often implicated in tissue-specific functions, development, and disease resistance [41,42,43].

This study comprehensively elucidated alternative splicing in bovine LIV, MUSC, TEST, and OVA tissues, providing valuable insights into the molecular complexity of bovine tissues. The prevalence of alternative exon skipping and differential splicing events across various organs underscores the importance of alternative splicing in regulating tissue-specific gene expression and function. The observed differences in splicing patterns between reproductive and non-reproductive organs highlight the role of alternative splicing in shaping reproductive processes. The higher number of differential splicing genes in the TESTs compared to the other tissues suggests the significance of alternative splicing in regulating male reproductive functions, such as androgen metabolism and spermatogenesis. Conversely, differences between male and female tissues, particularly those observed in the OVA, suggest the presence of critical gender-specific splicing patterns crucial for female reproductive processes.

In addition, we also found that TESTs have the greatest number of genes with APA events; this further confirms the complexity of transcription for testicular tissue. A previous study found that TEST-expressed APA displayed a lower incidence of AAUAAA, contained unique upstream and downstream sequence elements, and had shorter 3′ UTRs [44,45]. APA was also affected by alternative splicing in male germ cells, especially during the transition through meiosis [46,47]. Cytoplasmic chromatoid bodies are centers of multiple RNA metabolic processes in male germ cells [48]. In addition, a more significant number of DETs and DEGs between the TESTs and the other three tissue types were identified, all indicating the complexity of this tissue. The complexity of TEST functions is underpinned by the unique and specific gene expression profile, as indicated by the *TEX* gene family numbers, all of which were transcribed several times and highly expressed in the TESTs. The complexity of TSGs is regulated by a multitude of factors and mechanisms, as indicated by the annotation of numerous CGO, eggNOG, GO, KOG, and KEGG terms or pathways.

### 4.2. Exploring Testicular-Specific Expression Genes by Comparing the Gene Expression of Four Tissues in Bovines

Among the identified transcripts, 52.20% were novel. A recent study revealed that 61% of transcript isoforms were extremely TEST-specific, and TESTs exhibited the most complex transcriptome compared with those of the other tissues examined [16]. These novel transcripts might regulate spermatogenesis, as adult cattle were used in this study. The regulation of spermatogenesis involves expressing numerous genes in a precise cell- and stage-specific program [49]. More than 2300 genes are predominantly expressed in the mouse TEST, hundreds of which might facilitate the normal functioning of the male reproductive system or contribute to infertility in males [50,51]. 

Our study similarly unveils the complexity and significance of the TEST, with a significantly higher abundance of tissue-specific genes and transcripts in the TEST compared to the other tissue types, highlighting its pivotal role in reproductive processes. Enrichment of TEST-specific genes and transcripts in biological processes related to spermatogenesis, cellular scaffolding, and energy metabolism underscores the importance of the TEST in normal reproductive function. Interestingly, the study identifies numerous novel genes and transcripts, some of which may play crucial roles in regulating testicular function and spermatogenesis. The discoveries of these new genes offer new avenues for further research into male reproduction and may help address challenges in bovine herd reproduction. While many TEST-specific genes have been functionally validated in murine and human spermatogenesis, such studies are relatively scarce in the field of livestock. Furthermore, there remain many genes/transcripts whose functions have yet to be fully validated. The identification of novel TEST-specific genes/transcripts provides an opportunity for further functional studies, deepening our understanding of testicular function in cattle.

### 4.3. Identification of TEX Gene Transcripts and Prediction of Their Testicular-Specific Transcription Factors

Furthermore, certain highly expressed TSGs, such as TEXs, were focused on. The term “*TEX*” gene was coined by Wang et al. (2001) after they used cDNA suppression subtractive hybridization to explore new transcripts that were detected only in purified mouse spermatogonia [52]. However, little is known regarding their function [53]. *TEX* orthologs have also been found in other vertebrates (mammals, birds, and reptiles), invertebrates, and yeast [4,54]. To date, 69 *TEXs* (61 human and 61 mouse) have been identified in various species and tissues [4]. Herein, the expression of each *TEX* gene in cattle was also checked, with 224 *TEX* transcripts and 32 DEGs being detected. Additionally, 32 transcripts were identified for *TEX51*. A study of the genetic association between obesity and the *TEX51* [55,56] neuroendocrine disorder-related candidate gene suggested a lack of any correlation between *TEX51* and maternal obesity and reported that the highest number of transcripts of any *TEX* was recorded for *TEX51*, indicating the potentially critical function of this gene in male cattle reproductively. Hence, in-depth functional studies are warranted in the future. The expression of *TEX14* was also detected in other tissues. The TEST-enriched genes were ascertained to have novel functions and to be indispensable for male reproduction using an in vivo approach [57]. For instance, *TEX14* was essential for forming intercellular bridges and fertility in male mice [58] and might function similarly in cattle. In addition, our predicted potential transcription factors of *TEXs* that are highly expressed in the TEST, including CREB1, RUNX2, KLF5, and SOX5, are of great value in verifying their key regulatory roles and mechanisms in testicular spermatogenesis and male reproductive function when conducting functional experiments. 

*TSSK1B*, *TSSK6*, *DAZL*, *TSPYL6*, *TSSK4*, and *POLL* were highly expressed in the TEST and determined to be involved in various pathways, including “reproduction” and “reproductive process”. For example, *TSSK1B* played a pivotal role during spermatogenesis and was associated with male fertility and was explicitly expressed in yak TESTs and highly expressed upon sexual maturity [59]. The regulatory mechanisms of *TSSK1B* in male yaks require further study. Future research could focus on further improving the accuracy of the technology, developing more sophisticated bioinformatics tools for analyzing long-read data, and expanding their applicability to functional genomics. In addition, the function of the novel transcripts is largely unexplored; hence, further studies are needed, especially in the TEST. Moreover, we identified TEST-specific highly expressed transcription factors, including CREB1, RUNX2, KLF5, and SOX5, as transcription factors for testicular extracellular vesicle (*TEX*) genes. This identification holds significant reference value for further research into TEST-specific transcriptional regulation aiming to elucidate the unique functions of the TEST.

## 5. Conclusions 

Our findings revealed that the TEST exhibited the highest number of alternative splicing events, indicating a significant complexity in gene expression that is closely associated with its diverse functions. The identification of novel genes and transcripts in the TEST is crucial for future functional studies aimed at elucidating the molecular mechanisms of spermatogenesis. Our analysis of the *TEX* gene family confirmed their specific expression in the TEST and predicted TEST-specific transcription factors. This study underscores the complexity of gene expression and alternative splicing in cattle TESTs. Future research into the molecular regulatory mechanisms of TEST-specific gene expression and alternative splicing could provide valuable insights for improving cattle fertility. This could ultimately promote cattle reproduction, safeguard reproductive health, and increase cattle productivity. 

## Data Availability

The raw data supporting the conclusions of this article will be made available by the authors on request.

## Supplementary Materials

The following supporting information can be downloaded at: https://www.mdpi.com/article/10.3390/ani14111646/s1, Figure S1: Function annotation of differentially expressed transcripts (DETs) between each tissue and testis in cattle using CGO, eggNOG and KOG database; Figure S2: Analysis of differentially expressed transcripts (DETs) between each tissue and testis in cattle; Figure S3: The GO analysis diagram of the DETs between each tissue vs. testis in cattle and molecular functions are indicated; Figure S4: Function annotation of differentially expressed genes (DEGs) between each tissue and testis in cattle using CGO, eggNOG and KOG database; Figure S5: Analysis of differentially expressed genes (DEGs) between each tissue and testis in cattle; Figure S6: The GO analysis diagram of the DEGs between each tissue Vs. testis in cattle and molecular functions are indicated; Figure S7: Pathway enrichment analysis of testis specifically expressed genes/transcripts; Figure S8: Pathway enrichment analysis of transcription factors specifically expressed in bovine testes; Figure S9: Protein interaction diagram of bovine transcription factors. Table S1: Clean Data of 18 samples; Table S2: Full length sequence data statistics table; Table S3: Function annotation of novel transcripts; Table S4: Statistical table of variable splicing events; Table S5: SSR statistics; Table S6: comparison between full-length reads and known transcripts of the reference genome; Table S7: Number of differentially expressed transcripts (FDR = 0.01 FC = 1.5); Table S8: Number of differentially expressed mRNA.
