# Supplementary material for: A Comparative Full-Length Transcriptome Analysis Using Oxford Nanopore Technologies (ONT) in Four Tissues of Bovine Origin
Source: Animals (Basel). 2024 May 31;14(11):1646. doi: 10.3390/ani14111646 (PMC11170998; doi:10.3390/ani14111646)
Supplement: Supplementary file 1 [file animals-14-01646-s001.zip › Supplementary Figures-2024.5.24.pdf]

Supplementary figures

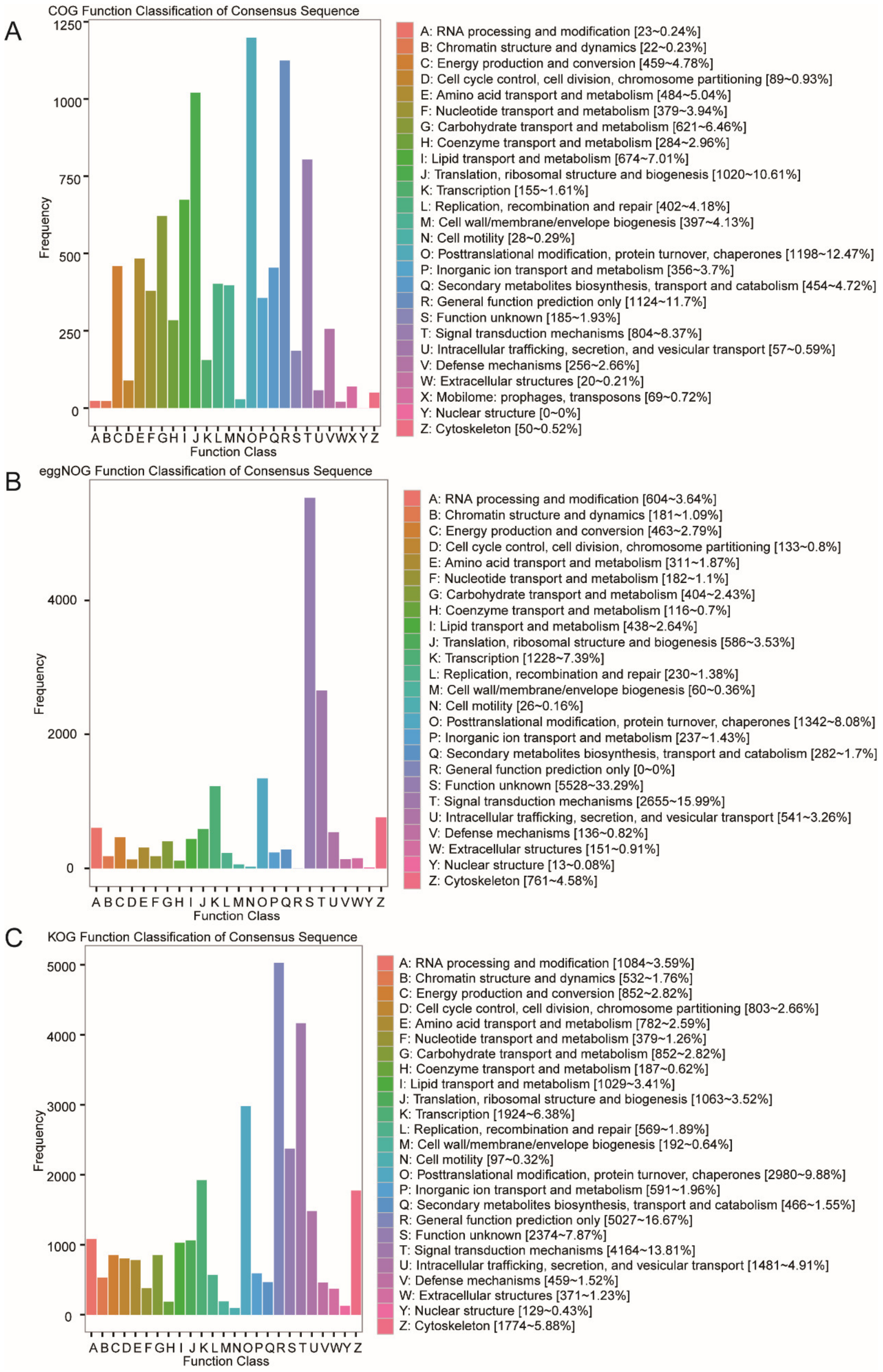

Figure S1. Function annotation of differentially expressed transcripts (DETs) between each tissue and testis in cattle using CGO, eggNOG and KOG database.

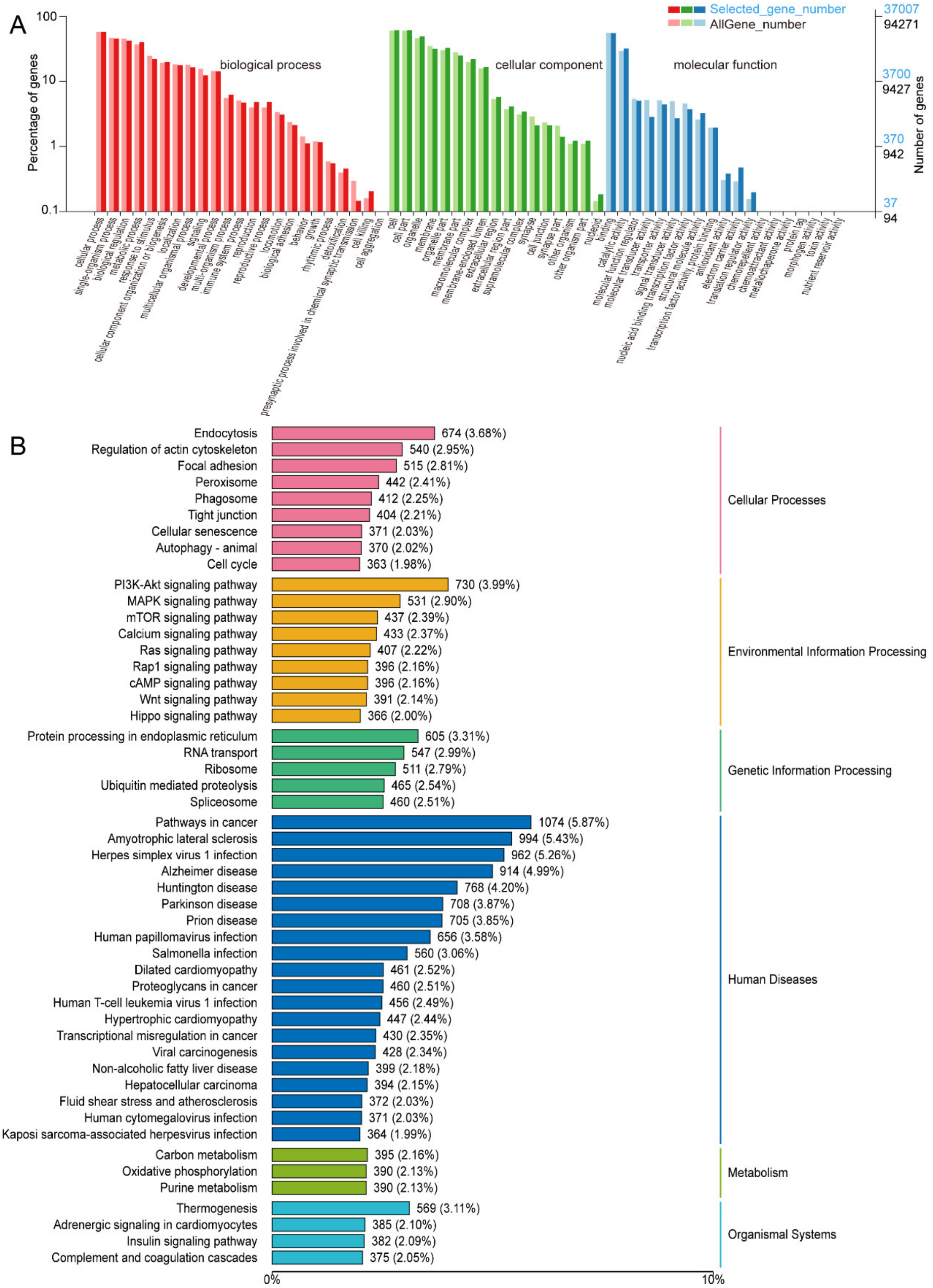

Figure S2. Analysis of differentially expressed transcripts (DETs) between each tissue and testis in cattle. (A) GO class annotation. (B) The KEGG class annotation.

C

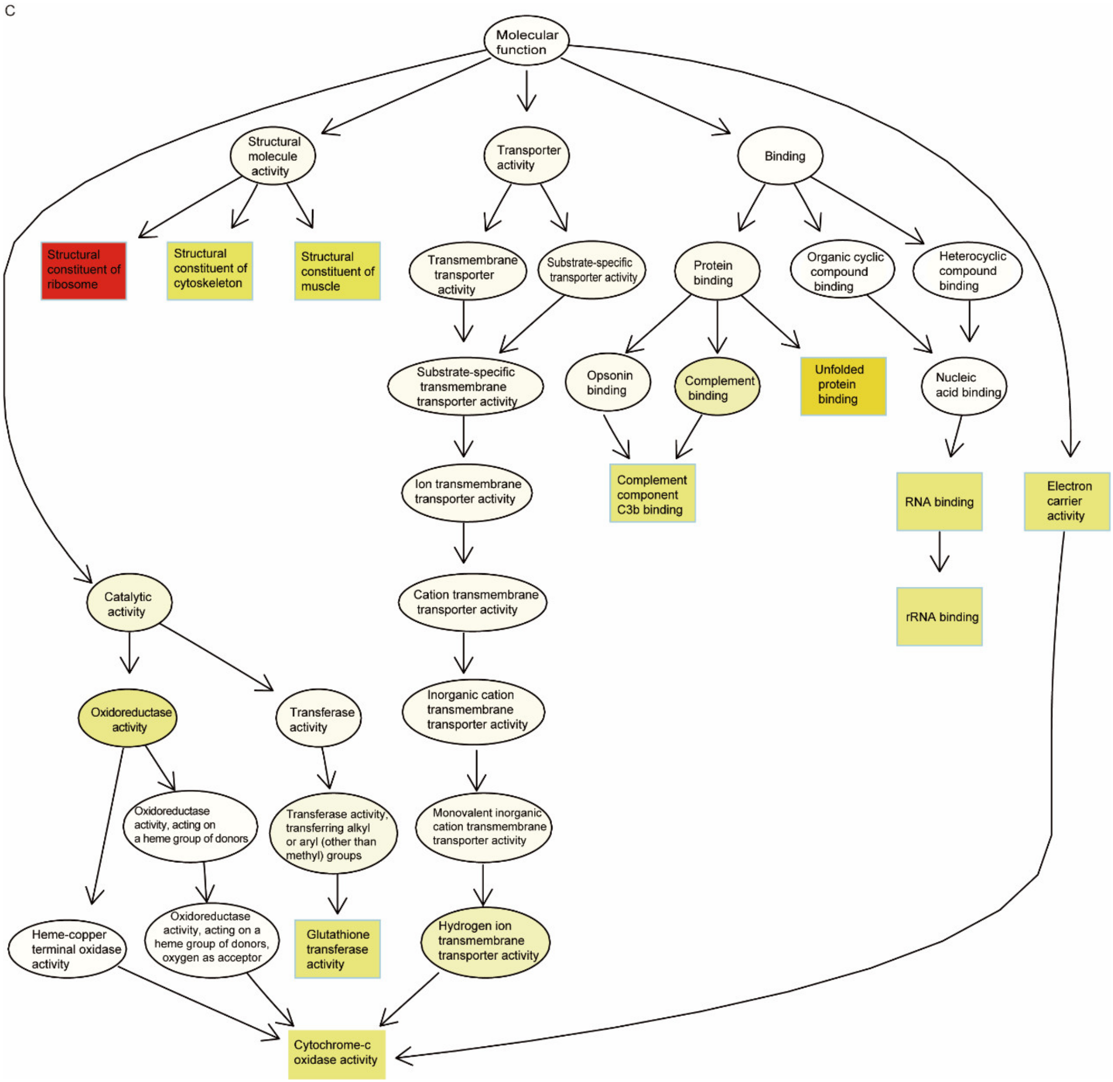

Figure S3. The GO analysis diagram of the DETs between each tissue Vs. testis in cattle and molecular functions are indicated.

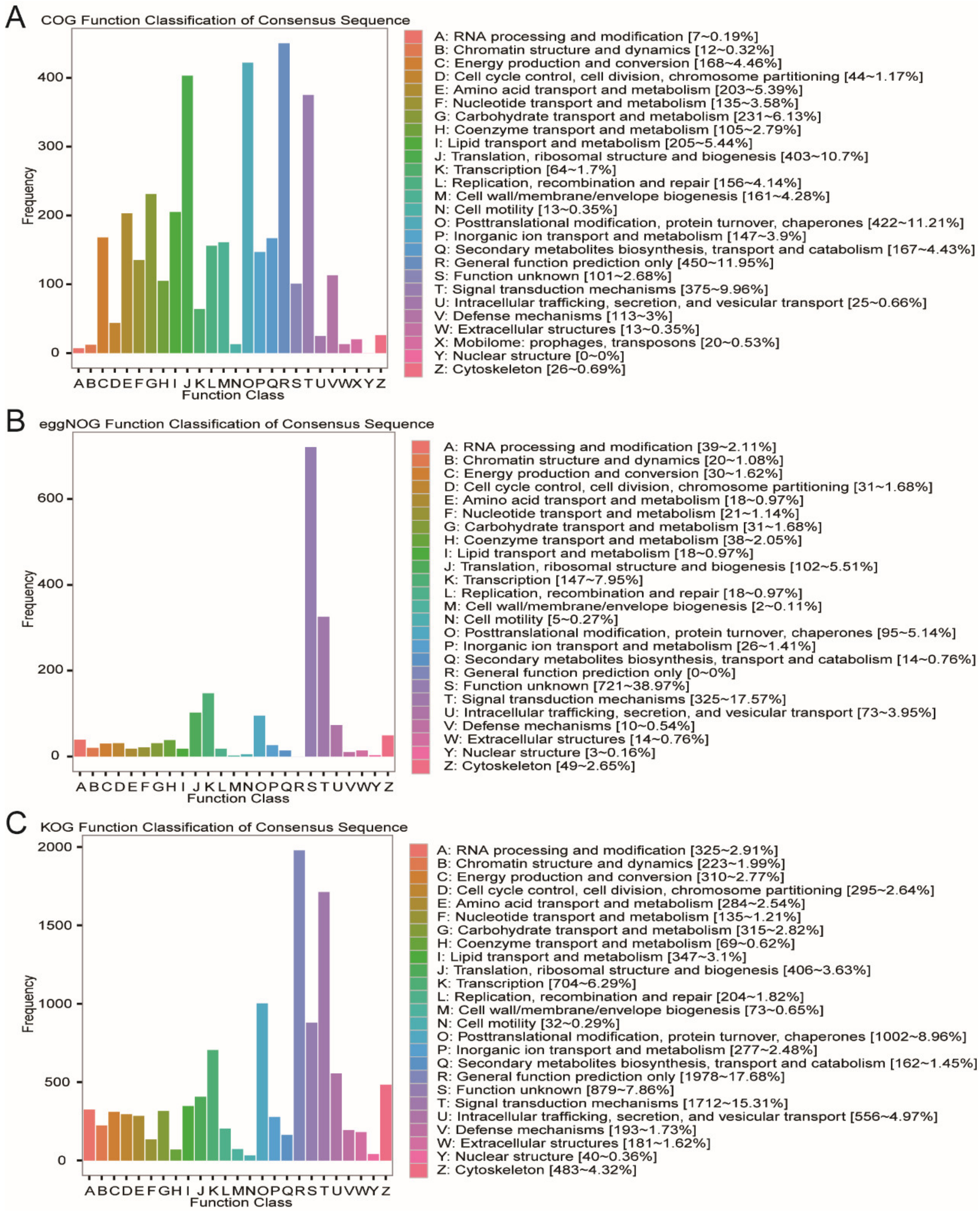

Figure S4 Function annotation of differentially expressed genes (DEGs) between each tissue and testis in cattle using CGO, eggNOG and KOG database.

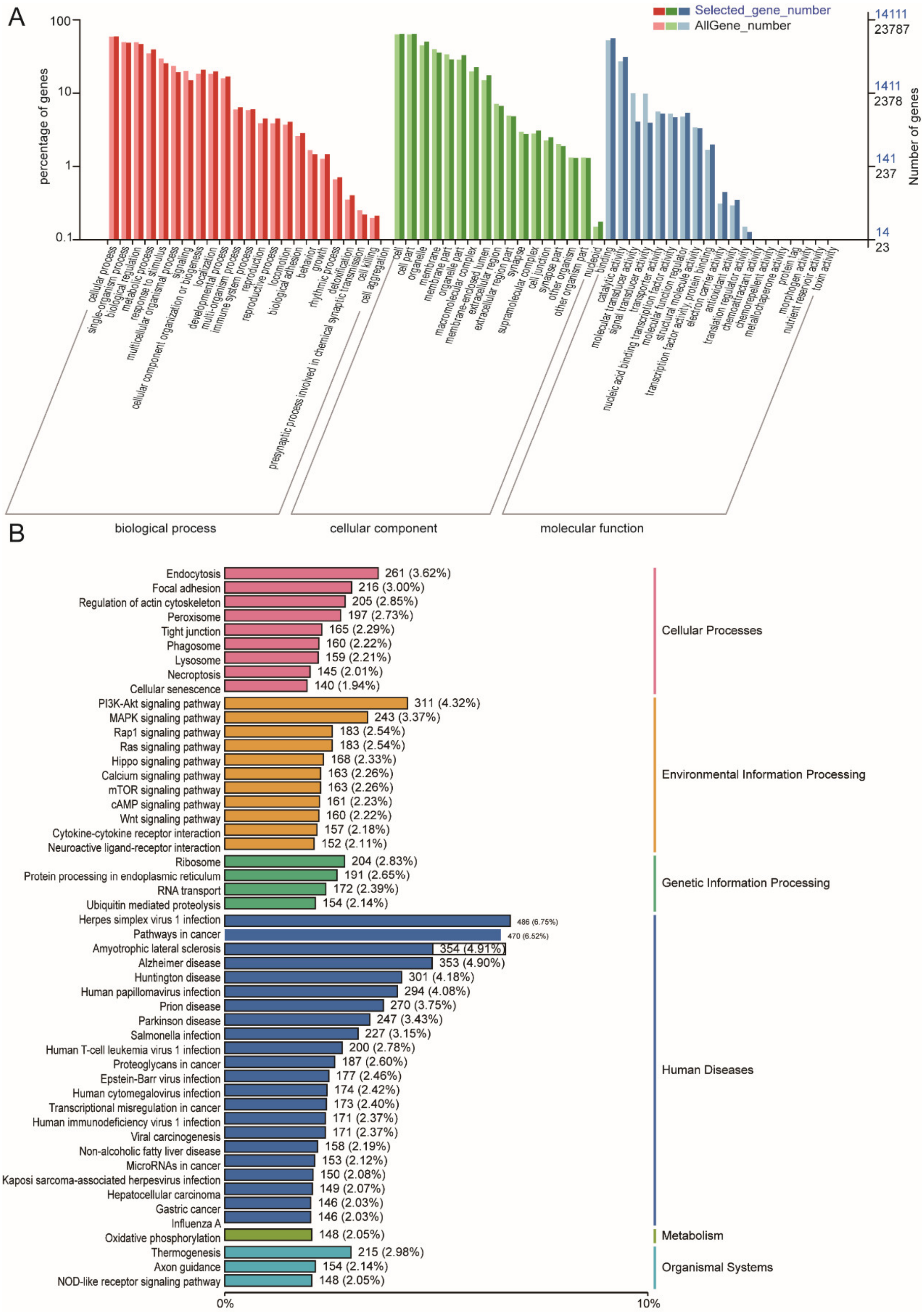

Figure S5. Analysis of differentially expressed genes (DEGs) between each tissue and testis in cattle. (A) GO class annotation. (B) The KEGG class annotation.

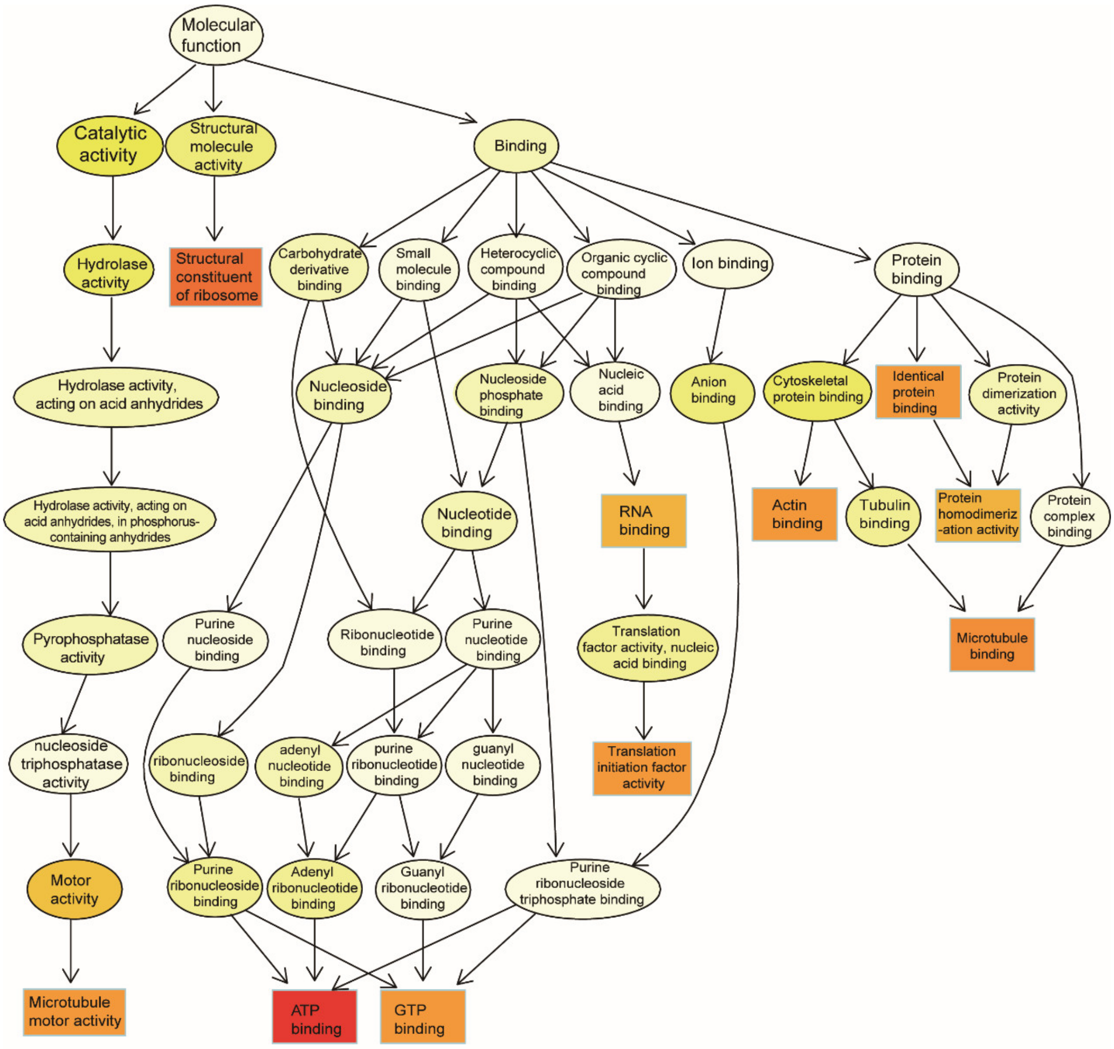

Figure S6. The GO analysis diagram of the DEGs between each tissue Vs. testis in cattle and molecular functions are indicated.

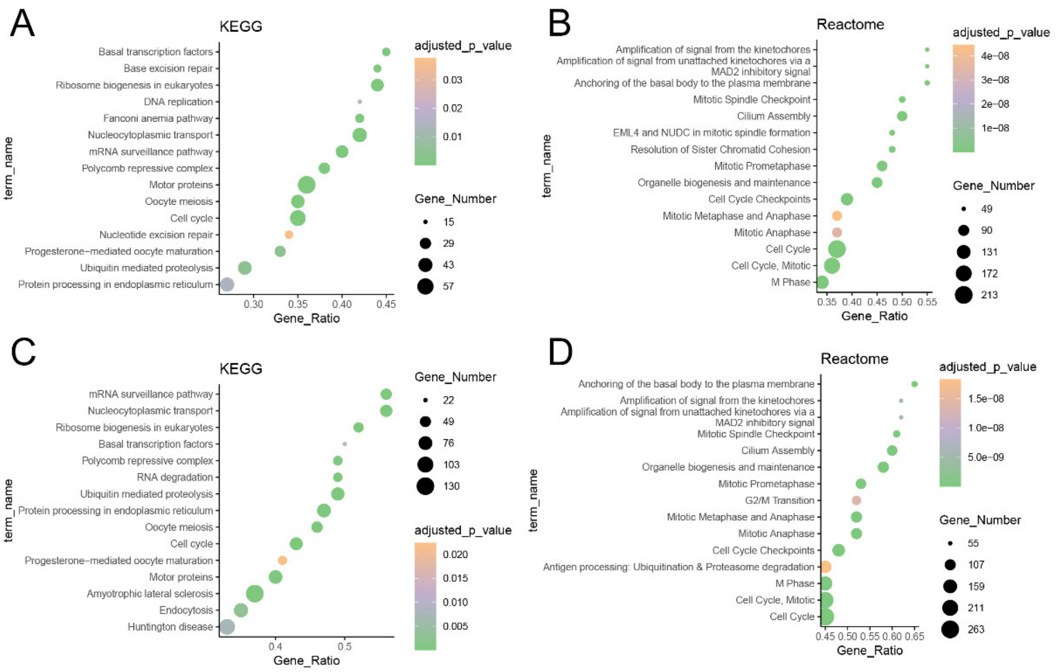

Figure S7. Pathway enrichment analysis of testis specifically expressed genes/transcripts. (A-B) The KEGG (A) and Reactome (B) enrichment of testis specifically expressed genes. (C-D) The KEGG (C) and Reactome (D) enrichment of testis specifically expressed transcripts.

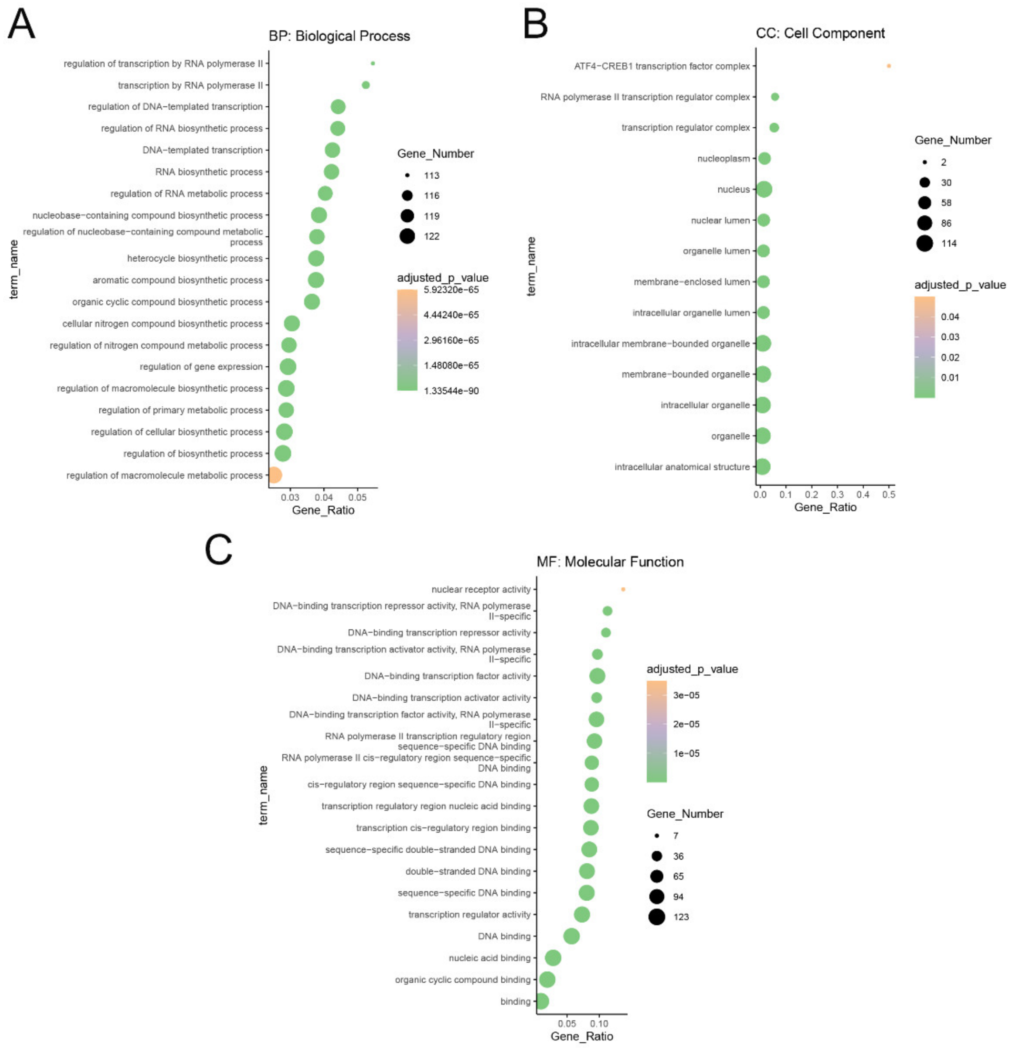

Figure S8. Pathway enrichment analysis of transcription factors specifically expressed in bovine testes.

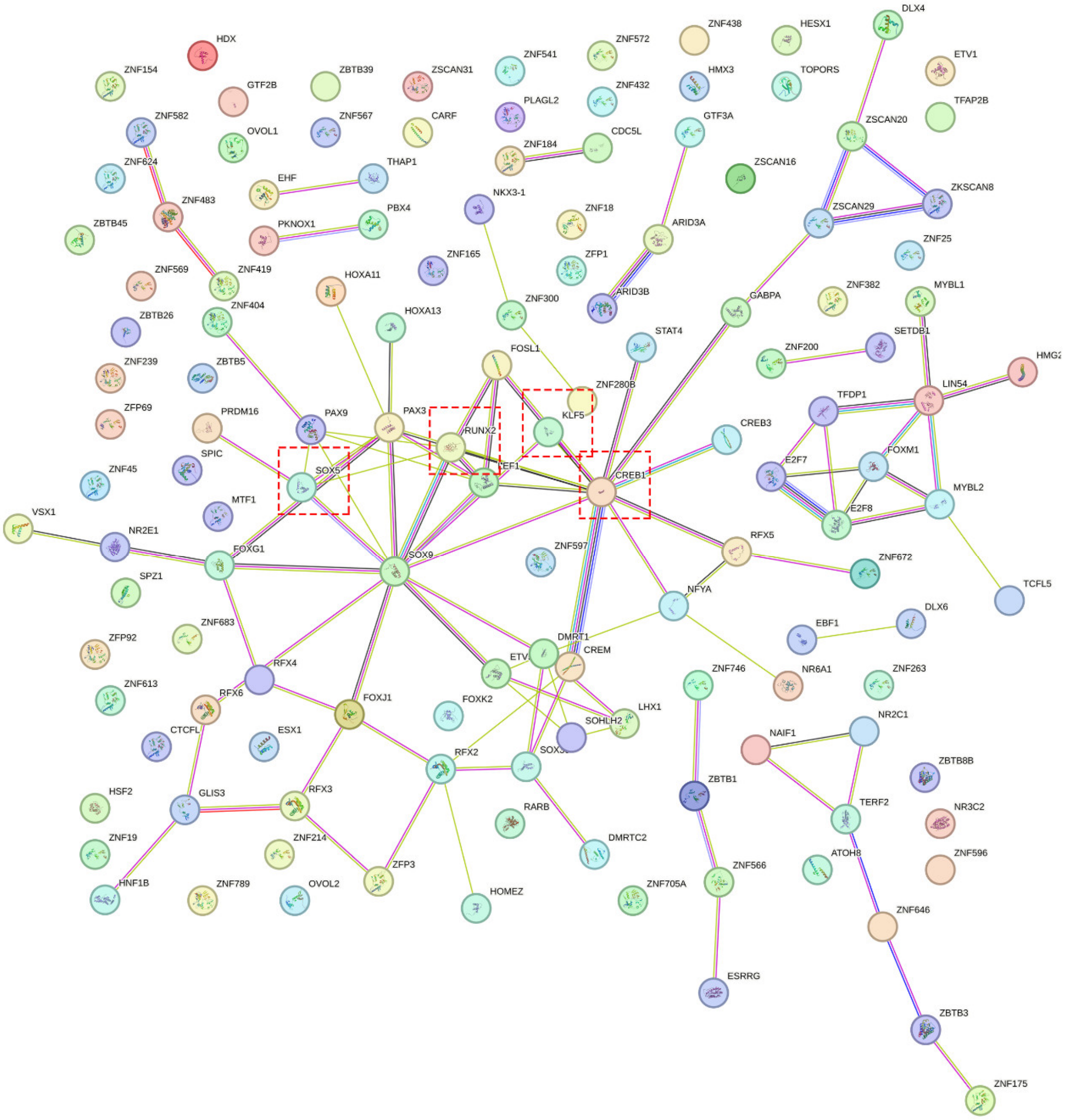

Figure S9. Protein interaction diagram of bovine transcription factors. The red dashed box represents the transcription factors of the *TEXs* family genes with high expression in cattle testes.
